# Pediatric Medical Traumatic Stress (PMTS) following Surgery in Childhood and Adolescence: a Systematic Review

**DOI:** 10.1007/s40653-021-00391-9

**Published:** 2021-08-21

**Authors:** Anna Stanzel, Susan Sierau

**Affiliations:** 1 Department of Clinical Child and Adolescent Psychology, Institute of Psychology, Leipzig University, Leipzig, Germany; 2 Department of Medical Psychology and Medical Sociology, Leipzig University, Leipzig, Germany

**Keywords:** Children, Externalizing, Hospitalization, Internalizing, Operation, Postoperative, Posttraumatic stress, Psychological symptoms, Surgical procedure, Youth

## Abstract

The purpose of the present review was to systematically review, synthesize and quantify prevalence rates of subclinical and clinical psychological symptoms in children and adolescents who have undergone surgery. Systematic literature searches were conducted twofold in April 8, 2020 and March 7, 2021 in PsycInfo and PubMed in accordance with the Preferred Reporting Items for Systematic Reviews and Meta-Analyses guidelines. Manuscripts were screened against eligibility criteria and were included if they investigated psychological symptoms in children or adolescents (age between 1 to 21 years at the time of study) who were hospitalized for pediatric surgery. Means and standard deviations weighted by sample size were calculated. Eleven articles met inclusion criteria. The review revealed that a small but substantial number of children and adolescents are at an elevated risk for postoperative psychological symptoms and disorders. Up to 13% had symptoms consistent with a diagnosis of a posttraumatic stress disorder, 6% to 8% exhibited elevated symptoms of depression or anxiety, and about 25% showed internalizing and externalizing symptoms. This review provides preliminary evidence that children and adolescents experience significant psychological distress and posttraumatic stress symptoms after pediatric surgery. Apart from better training of clinicians, prevention, early psychological screening and psychosocial care in surgical wards of hospitals are recommended. Well-designed studies of high methodological quality are necessary to replicate existing findings and provide a broader base of evidence.

**Supplementary Information:**

The online version contains supplementary material available at 10.1007/s40653-021-00391-9.

## Introduction

Traumatic injury, critical illness or other medical conditions entail prolonged stays in the pediatric intensive care unit (PICU) and hospitalizations accompanied by emergency or elective surgery.^1^ Children and adolescents hospitalized for surgery are exposed to potentially traumatizing situations such as separation from parents, unfamiliar surroundings with frightening equipment and surreal technology, pain from invasive procedures and mental dizziness due to shock or anesthesia (Forgey & Bursch, 2013). As a result children are at risk for chronic physical and mental health issues (Als et al., 2015) and psychological functioning may be impaired (Ben-Ari et al., 2018). Recent decades have witnessed advances in medical technology and postoperative care (Hung et al., 2018) resulting in an increase in success and survival rate of surgery (Brown et al., 2015). Nevertheless, children and adolescents may suffer psychological consequences of their medical treatment. Accompanying this recent development, a systematic review on psychological sequelae in children and adolescents after surgery was conducted.

Awareness of posttraumatic stress disorder (PTSD) symptoms following medical trauma has increased over the last 15 years: While 2005 only 7% of physicians recognize that children could develop posttraumatic stress symptoms (PTSS) after traffic-related injury (Ziegler et al., 2005), the majority of emergency department (ED) staff is aware that an injured child is at risk for traumatic stress in 2017 (Hoysted et al., 2017). However, adequate knowledge of PTSD and the skills to recognize and treat PTSD is still lacking among ED staff (Banh et al., 2008; Hoysted et al., 2017, 2018; Kassam-Adams et al., 2015a). In a worldwide survey, less than half of the ED staff (45%) answered knowledge questions regarding psychosocial care for injured children correctly (Alisic et al., 2016). Negative sequelae are associated with non-adherence to medical care and avoidance of follow-up treatment (Shemesh et al., 2000). Screening for negative sequelae fosters early identification and referral to psychosocial care (Kazak et al., 2011). Although undetected and untreated PTSS pose a threat to adequate medical care and full recovery (Broadbent et al., 2012; Shemesh et al., 2000), Moss et al. (2019) reported “screening” being still the least frequently observed practice.

### Pediatric Medical Traumatic Stress

Pediatric medical traumatic stress (PMTS) is defined as “a set of psychological and physiological responses of children and their families to pain, injury, serious illness, medical procedures and invasive or frightening treatment experiences” (National Child Traumatic Stress Network [NCTSN], 2018). These reactions refer to, but are not limited by, PTSS, including reexperiencing, avoidance of trauma-related stimuli, hyperarousal, and negative alterations in cognitions and mood as defined by the newest edition of the Diagnostic and Statistical Manual of Mental Disorders (DSM-5; American Psychiatric Association [APA], 2013). The present review follows that approach and is inclusive to more than just fulfilling a clinical diagnosis following a medical event. Additionally, other psychological symptoms after the surgery, including depression, anxiety, and behavioral problems are considered.

Medical events are considered to be *potentially* traumatic since the same objective event is not uniformly traumatogenic (Kazak et al., 2006). On the one hand, the length of hospitalization in PICU, seriousness of illness and number of procedures was found to relate to PTSS (Connolly et al., 2004; Rennick et al., 2002). On the other hand meta-analyses suggest that objective characteristics have poor predictive value, while perceived life-threat is a strong predictor of trauma symptoms (Cox et al., 2008; Trickey et al., 2012). In a recent meta-analysis more severe illnesses as well as longer and more intense treatments were clearly related to higher PTSS (Pinquart, 2020).

In the aftermath of the medical trauma children commonly experience acute stress symptoms and transient emotional distress (Nelson et al., 2019; Winston et al., 2002). Initial acute distress is normative, helps to adapt, and resolves mostly spontaneously (Kazak et al., 2006). Thus, the majority of children are resilient to the traumatizing effect or can draw on sufficient protective factors (Le Brocque et al., 2020; Pinquart, 2020). A significant subset of children, however, is not able to adapt despite support, leading to persistent posttraumatic stress symptoms for at least 12 months (De Young et al., 2012; Le Brocque et al., 2020). On an individual level, for children diagnosed with PTSD, symptoms remain elevated beyond six months without intervention (Le Brocque et al., 2020).

### Prevalence Rates of Pediatric Medical Traumatic Stress

A meta-analytic study revealed 19% of injured children and 12% of medically ill children fulfill diagnostic criteria for PTSD (Kahana et al., 2006). Pinquart (2020) updated and extended this first meta-analysis and found a similar rate of 11.5% of individuals with pediatric chronic physical illnesses meeting the criteria for PTSD. Further, PTSS were more prevalent in this population compared to healthy peers. Prevalence rates of PTSD range from 10 to 20% and rates of PTSS range from 25 to 30% among medially ill children (Forgey & Bursch, 2013). About one third of severely burn-injured children develop acute and posttraumatic stress symptoms (Bakker et al., 2013). In the first four months after motor vehicle accidents, PTSD is diagnosed in 12% to 46% of children and adolescents (Mehta & Ameratunga, 2012). The incidence of moderate to severe PTSS was higher following accident-related injuries (14.6%) than for other serious pediatric health conditions such as cancer (10.0%) or diabetes (5.4%; Landolt et al., 2003).

Owing to the more inclusive conceptualization of PMTS, only looking at PTSS at both diagnostic (PTSD) and subsyndromal levels (PTSS) may underestimate the true rates of PMTS. A comprehensive review after pediatric critical illness reported point prevalence as high as 10% to 28% for PTSD and ranging from 7 to 13% for depressive symptoms (Davydow et al., 2010). Over the course of one year following discharge after meningococcal disease, 25% of children developed depression, 10% developed oppositional defiant disorder, 7% developed adjustment disorder, and 7% developed anxiety disorder (Shears et al., 2007). In an intervention study, 25.9% of critically ill children in the control group displayed behavioral problems whereas only 2.3% of the intervention group (Melnyk et al., 2004). PTSD rates in psycho-oncology research range from 3 to 18%, and elevated levels of anxiety were reported by adolescents (Kazak et al., 2004; McDonnell et al., 2017).

Mental health consequences of medical trauma are heterogeneous. After emergency medical treatment for an injury, 15% and 13% had significant PTSS and depressive symptoms, respectively (Kassam‐Adams et al., 2015b). Depression and PTSS occur highly comorbid, e.g., 17% of hospitalized injured adolescents reported high levels of both (Zatzick et al., 2008). For young burn-injured children, prevalence rates of 25% for PTSD and 16% each for oppositional defiant disorder and separation anxiety disorder were reported (De Young et al., 2012). The pediatric research examining psychiatric morbidity in survivors of PICU has focused on trauma-related stress symptoms but not in-depth on depressive, anxiety and psychotic symptoms (Davydow et al., 2010).

### The Present Study

While the main focus of pediatric trauma research has been on the PICU population following admission for critical illness or traumatic injury and, more specifically, burn-injured children, victims of motor vehicle accidents, or cancer patients, pediatric trauma comes in many forms. One understudied area of pediatric medical trauma is the surgery population. To date, there has been no systematic review conducted focusing on PMTS after surgery. Studies examining children hospitalized for various medical treatments limit the ability to draw conclusions on psychological consequences of specific treatments or characteristics of their hospital stay.

The rationale of the present review is to identify which subclinical or clinical symptoms of psychological disorders were reported among children and adolescents after surgery and how many children and adolescents develop subclinical or clinical symptoms of psychological disorders after surgical procedures. The aim of this study is twofold: (1) Descriptive information along with prevalence rates of psychological symptoms and disorders in children and adolescents who were exposed to surgical procedures are synthesized. Additionally, (2) a quantitative analysis is conducted to identify common symptoms in children and adolescents having experienced surgical procedures.

## Methods

This systematic review is following the Guidelines defined by Preferred Reporting Items for Systematic Reviews and Meta-Analyses (PRISMA; Moher et al., 2009). A protocol for this review was not registered before conducting the review.

### Search Strategy

The electronic literature search was conducted in PsycInfo and PubMed on March 7, 2020 and was updated on April, 8 2020. Under the assumption that medical trauma is a relatively understudied area the search was conducted as inclusive as possible. A second search focusing on pediatric surgery was performed on March 6, 2021 in PubMed only. Search terms used in both databases are outlined in Table 1 and limiters applied in detail are provided in Table S1 (see Supplementary Material).

**Table 1:** Database Search Terms

Category	Search Terms and Boolean Operators	
	Search March 7, 2020 and April 8, 2020	Search March 6, 2021
Medical event	("pediatric injury" OR "medical illness" OR pediatric OR "medical procedure*" OR "medical treat*" OR "intensive care") AND	("surgery" OR "surgical" OR "operative") AND
PMTS	("posttraumatic stress" OR "posttraumatic syndrome" OR "post-traumatic stress" OR "post-traumatic syndrome" OR "pediatric medical traumatic stress" OR "medical trauma") AND	
Impact	("mental health" OR "psychological symptom*" OR "psychiatric disorder" OR interna* OR externa* OR depress* OR anxi* OR emotional OR behavi* OR "post-traumatic stress disorder")	

By using “*” variations of keywords were included in the search

Aside from the protocol-driven search approach, complementary searches were executed. References of relevant reviews and meta-analyses were searched for additional articles meeting eligibility criteria. Lastly, a manual search of references within relevant extracted articles, referred to as reference mining, was performed to identify additional papers.

### Study Eligibility Criteria

We aimed to identify studies in accordance with the following inclusion criteria: original research articles published in peer-reviewed journals; available as full-text in German or English; children or adolescents between birth and 21 years of age; reporting validity and reliability on instruments or using well-established instruments; assessment of (sub-) clinical psychological symptoms any time following a potentially traumatic medical event; and empirical quantitative data. Psychological symptoms include PTSS/ PTSD, depressive and anxiety symptoms, and internalizing and externalizing symptoms. Studies assessing surgical procedures were included. In case of duplicate publications of the same study sample, only the study with the most complete and relevant information was included. No restriction was placed on the publication period or the study design.

Studies were excluded if they were reviews, meta-analyses, case studies, book chapters, abstracts, protocol studies, dissertations, theses, editorials, qualitative studies, letters to the editor, and clinical guidelines. We excluded studies that reported data solely on family members and caregivers of affected pediatric participants. Other studies that reported psychological outcomes such as self-esteem, body image, broader functional outcomes and quality of life were excluded. Cancer populations were excluded because pediatric psycho-oncology is a distinctive research field itself. Transplant surgery was excluded due to concerns for the confounding of risk for psychiatric morbidity conferred by organ transplants owing to psychiatric adverse effects of immunosuppressant medications (Heinrich & Marcangelo, 2009) and the severity of chronic illness before the transplant (Olbrisch et al., 2002). Head injuries were deliberately excluded from this review since brain-organic damage and the resulting psychopathological consequences cannot be clearly delineated (e.g., cognitive problems after traumatic brain injury are associated with behavioral difficulties; Coulter & Forsyth, 2019).

### Study Selection

**Fig. 1:**
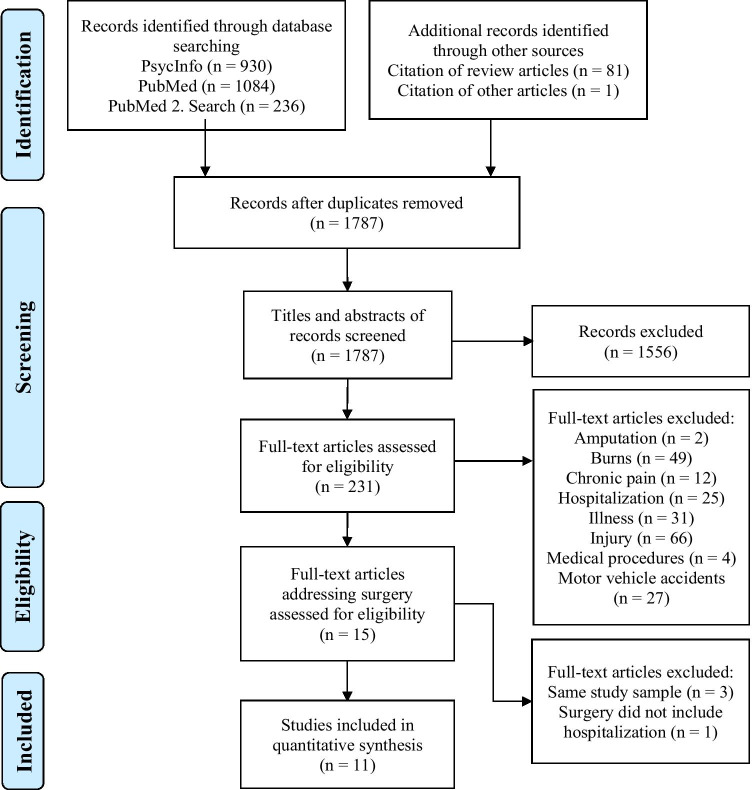
PRISMA 2009 Flow Diagram

All study records revealed through the electronic literature search were uploaded into Citavi 6, an electronic bibliographic software to be organized and managed comprehensively by the first author. After doublets were removed, titles and, if needed, abstracts of all study records (n = 1787) were screened to assess their eligibility. Full-texts of potentially relevant studies (n = 231) were retrieved for screening against inclusion and exclusion. Excluded studies were assigned into categories providing a preliminary overview (see Fig. 1). Due to the research gap, the area of surgery was examined and 15 related studies were identified. If there was a question about whether to include a study or not, both reviewers assessed the relevant studies and differences were resolved by discussion. The study selection process led to the inclusion of 11 articles while four were excluded (see Fig. 1).

### Data Extraction

One author completed data extraction using a constructed Microsoft Excel spreadsheet while the second author verified the extracted data. Relevant data extracted included (1) general study characteristics (name of the first author, year of publication, the country where the study was conducted); (2) sample characteristics (size, ages); (3) study design; (4) information on surgical procedures; (5) outcomes, instrument and time of assessment; (6) prevalence of outcomes; (7) main results, as well as prevalence rates. The most relevant information is presented in the Results in Table 2. One study author was contacted in order to receive relevant data for inclusion in quantitative synthesis. The respective data was received.

**Table 2:** *Characteristics and Main Results of Studies Included in the Analysis of the Review*

First Author Year Country	Sample Characteristic	Design ^c^	Surgical Procedure	Outcome (Instrument)	Time of Assessment	Main Result and Prevalence		Risk of Bias ^d^
Ben-Amitay et al. (2006) Israel	*N* = 40 (52.5% boys); ages 6 – 18 (*M* = 13.05, *SD* = 2.7)	P	elective surgery	PTSD (CPTS-RI) Depressive symptoms (CDI) Anxiety symptoms (RCMAS)	Admission day (Stage 1) 1 month after surgery (Stage 2), 6 months after surgery (Stage 3)	PTSS (7.7% mild), depressive symptoms (5%) were low and decreased over time. Anxiety symptoms (12.5%) were low, but did not change over time. (Data based on Stage 2)		7
Ben-Ari et al. (2018) Israel	*N* = 79 (65% boys); ages 1 – 6 (*M* = 4.5, *SD* = 2.8)	P	minor surgery: 50%, other kinds of surgery: 32%, without surgery 15%, burns: 3%	PTSD (PTSDSSI, PCASS) Internalizing and externalizing symptoms (CBCL)	3 to 5 months post-discharge	10.39% of the children exhibit posttraumatic symptoms. In the CBCL’s general scale 11% of children were in the clinical and 4% in the borderline range. Internalizing problems were reported the most		6
Ben-Ari et al. ^a^ (2019) Israel	*N* = 30 (47% boys); ages 1 – 13 (*M* = 4.23, *SD* = 3.1)	P	surgery for congenital melanocytic nevi	PTSD (PTSDSSI, PCASS, UCLA-PTSD-RI-P) Internalizing and externalizing symptoms (CBCL)	Before hospitalization, 4 months post-discharge	33.3% of the children met PTSD diagnosis In the CBCL’s general scale 26.7% of children were in the borderline range and 30% in the clinical range. Internalizing problems were reported the most		7
Ben-Ari et al. ^b^ (2019) Israel	*N* = 88 (58% boys); ages 6 – 13 (*M* = 9.03, *SD* = 2.3)	P	surgery: 80.7%, without surgery: 19.3%	PTSD (UCLA-PTSD-RI-P) Internalizing and externalizing symptoms (CBCL)	Baseline, 3 to 5 months post-discharge	10.2% of the children fully met PTSD diagnosis, whereas 26.4% met partial diagnosis In the CBCL’s general scale 27.3% of children were in the clinical range		7
Connolly et al. (2004) USA	*N* = 43 (60% boys); ages 5 – 12 (*M* = 8.2, *SD* = 2.5)	P	cardiac surgery	PTSD (DISC)	4 to 8 weeks post-discharge		12% of the children met diagnostic criteria for PTSD, and 12% had symptoms but did not meet criteria	6
DeMaso et al. (2014) USA	*N* = 139 age: *M* = 16.01, *SD* = 0.5	P	arterial switch operation	mood, anxiety and disruptive behavior disorders (K-SADS-PL)	16 years after operation as an infant		The most common psychiatric diagnosis was ADHD (16%) 4% had symptoms of mood disorder, 5% of anxiety disorder, and 16% of disruptive behavior disorder	7
Kubota et al. (2011) Japan	*N* = 72 (51.39% boys); ages 6 – 17	R	major neonatal surgery	Internalizing and externalizing symptoms (CBCL)	6 to 17 years after surgery as an infant		Among the subjects having the most typical neonatal surgical diseases requiring long-term follow-up 45% scored in the clinical range of the CBCL	5
Lopez et al. (2008) Switzerland	*N* = 7 (85.71% boys) ages 8 – 16	R	intraoperative awareness	PTSD (new questionnaire based on CPTS-RI)	8 to 15 months after surgery		None of the children demonstrated symptoms of PTSD (0%)	4
Phelan et al. (2009) Australia	*N* = 4 (25% boys); ages 6 – 10	R	intraoperative awareness	PTSD (TSCC-A)	4 to 5 years after surgery		None of the children were in the clinically significant range of any symptoms of posttraumatic stress (0%)	5
Sarrechia et al. (2015) Belgium	*N* = 48 (39.58% boys); Ages 6 – 12 (*M* = 9.25, *SD* = 1.75)	R	open heart surgery or catheterization	Internalizing and externalizing symptoms (CBCL)	several years after surgery (not stated explicitly)		In the CBCL’s general scale 3.8% of children were in the borderline range and 8.1% in the clinical range	5
Toren and Horesh (2007) Israel	*N* = 31 (61.29% boys); ages 10 – 21 (*M* = 14.7, *SD* = 0.3)	C	cardiac surgery	PTSS (UCLA-PTSD-RI) depressive symptoms (BDI) anxiety symptoms (SCARED)	13.7 (2.48) years after surgery		A significant portion of children undergoing cardiac surgery display PTSD symptoms (29.03%), depressive (18.52%) and anxiety (16.13%) symptoms	6

*N* sample size, *M* mean, *SD* standard deviation, *BDI* Beck Depression Inventory, *CBCL* Achenbach's Child Behavior Checklist, *CDI* Children's Depression Inventory, Conners' *ADHD* attention-deficit/hyperactivity disorder, *CPTS-RI* Child Posttraumatic Stress Reaction Index Revision, *DISC* Diagnostic Interview Schedule for Children, *K-SADS-PL* Schedule for Affective Disorders and Schizophrenia for School-Aged Children-Present and Lifetime Version, *PCASS* Preschool Children's Assessment of Stress Scale, *PTSD* posttraumatic stress disorder, *PTSS* posttraumatic stress symptom, *PTSDSSI* Posttraumatic Stress Disorder Semistructured Interview, *RCMAS* Revised Children's Manifest Anxiety Scale, *SCARED* Screen for Child Anxiety Related Emotional Disorders, *TSCC-A* Trauma Symptom Checklist for Children Alternate version, *UCLA-PTSD-RI* University of California at Los Angeles- Post Traumatic Stress Disorder Reaction Index

^a^Ben-Ari et al. 2019a

^b^Ben-Ari et al. 2019c

^c^P = prospective; R = retrospective; C = cross-sectional

^d^8 or 9 = low risk of bias; 7 = moderate risk of bias; 6 or less = high risk of bias

### Data Analysis

For each study that met inclusion criteria, the descriptive information including the mean age of children and adolescents, percentage of the sample that was male, percentages of children reported as having PTSD, partial PTSD, depression, anxiety, and disruptive behavior disorder symptoms were extracted as well as percentages of children scoring in the clinical or borderline range of the Achenbach’s Child Behavior Checklist (CBCL). Heterogeneous surgical events, the diversity of outcome measures, the age range included in this review, and the data itself did not allow a meta-analysis. A pooled analysis accounting for between-study heterogeneity requires the same study design and statistical models across studies and could, therefore, not be applied in the present review (Borenstein et al., 2010). Instead, weighted means and standard deviations were calculated.

### Risk of Bias

The risk of bias assessment was conducted in accordance with PRISMA. Even though the PRISMA method involves preferably using the Cochrane Risk of Bias Tool (Higgins et al., 2011), which measures risk of bias of intervention studies, an alternate measure specifically developed for assessing risk of bias in prevalence studies was used (Hoy et al., 2012). The Risk of Bias Tool (see Table S2 in the Supplementary Material) comprised 10 yes/no items, consisting of four measures of external validity (e.g., whether the sample was representative of the target population) and six measures of internal validity (e.g., whether the study instrument used to define a case was reliable and valid). For example, answering yes to Item 8 from the measure (“Was the same mode of data collection used for all subjects?”) indicates that the study was at low risk of bias for that item while answering “no” indicates a high risk of bias for that item.

One item assessing whether the sample was “a close representation of the national population” had to be omitted in our assessment of each study’s risk of bias. The target population for this review (pediatric surgery population) is known to be different from the national population and is not representative of a national sample. After omitting one of ten items a total number of nine items with the highest possible score of 9 was included. The same modification of the tool was made in a previously published systematic review (Cerimele et al., 2014). Studies with scores of 8 or 9 indicate that the study was at low risk of bias; studies with scores of 7 were considered to have moderate risks of bias, and studies with scores of 6 or less were categorized as having high risks of bias. If a study did not provide sufficient information in order to assess the risk of bias for an item, the answer to that item was considered “no” (i.e., the study is at risk of bias for that item).

### Open Data and Open Materials

All data files, associated codebooks, and analysis scripts are publicly available on the study’s page on the Open Science Framework: https://osf.io/vp9d8/

## Results

First, study characteristics are described (see Table 2). Further, results regarding each reported psychological symptom are synthesized. Prevalence rates of psychological symptoms are averaged and weighted by sample sizes (see Table 3).

**Table 3:** Demographics and Prevalence Rates Among Pediatric Surgery Samples of the Included Studies

Variable	Number of Studies	Weighted Mean ^a^	Weighted SD ^a^	Number of Studies reporting Prevalence Rates		
				0%	Below 15%	Above 15%
Percentage male	11	55.5%	8.99			
Age	11	10.6 years	4.38			
PTSD Symptoms	8	12.84%	9.7	3	3	2
Subthreshold PTSD Symptoms	3	18.4%	8.39	1	1	1
Depressive Symptoms	3	6.33%	5.1		2	1
Anxiety Symptoms	3	8.07%	4.43		2	1
Disruptive Behavior Disorder Symptoms	1	16%	NA			1
CBCL Clinical Range	5	24.61%	13.68		2	3
CBCL Borderline Range	3	8.31%	8.97		2	1

*CBCL* Child Behavior Checklist (assessing internalizing and externalizing symptoms), *PTSD* posttraumatic stress disorder, *SD* standard deviation

^a^weighted by sample size

### Characteristics of the Studies

For each of the 11 included studies, study and sample characteristics, the design, the surgical procedures, measurements of PMTS, the time of assessment, the main results with prevalence rates and the Risk of Bias score is reported (see Table 2). The included studies were published between 2004 and 2019. Most of the studies were conducted in Israel and the USA. The average sample size was 52.82 participants (*SD* = 39.35). All studies included both genders, and the age ranged from 1 to 21. The weighted mean age of participants was 10.6 years (*SD* = 4.38), and about half (55.5%) of the participants were male weighted by sample size. Among the studies, one was cross-sectional, four were retrospective, and six were prospective. Only two studies included a control group (DeMaso et al., 2014; Sarrechia et al., 2015).

Although the majority of the included studies used well-validated and reliable measures of PTSD, anxiety, depression and other psychological sequelae likely because of traumatic experience, only Ben-Ari and colleagues explicitly referred to the construct of PMTS (e.g. Ben-Ari et al., 2018). Since most studies assessed PTSD symptoms, they only referred to PTSS but not explicitly to PMTS. Eight studies reported on PTSD symptoms and five studies assessed internalizing and externalizing symptoms by means of the CBCL, mostly referred to as overall distress level. Only three studies assessed depressive and anxiety symptoms. One study also assessed disruptive behavior disorders, such as attention deficit hyperactivity disorder (ADHD) and others. Concerning time of assessment, most of the studies assessed three to five months post-discharge, whereas five studies made their assessment several years after the surgery. Studies involved cardiac surgery (n = 3), intraoperative awareness (n = 2), surgery for congenital melanocytic nevi (n = 1), arterial switch operation as infants (n = 1), major neonatal surgery (n = 1). Two studies included various surgical procedures in a pediatric surgery ward, resulting in a representative sample of participants. It has to be noted that the respective studies included each 15% to 20% participants hospitalized without surgery (Ben-Ari et al., 2018, 2019c). Solely elective surgery was subject of investigation in one study (Ben-Amitay et al., 2006).

### Prevalence Rates of Psychological Symptoms

Table 3 provides an overview regarding the number of studies reporting 0%, less than 15%, or more than 15% of the study sample displaying psychological symptoms. Figure S1 presents prevalence rates across included studies (see Supplementary Material).

#### Posttraumatic Stress Symptoms

Of the 11 included studies, eight studies examined symptoms of PTSD. Eight studies referred to PTSD at diagnostic level and three referred to posttraumatic stress symptoms at symptom level not meeting criteria (subthreshold PTSD or partial PTSD). PTSD prevalence rates ranged from 0.0% to 33.3%, with a weighted mean of 12.84% (*SD* = 9.7) averaged across eight studies weighted by sample size. Subthreshold PTSD prevalence rates ranged from 0.0% to 26.4%, with a weighted mean of 18.4% (*SD* = 8.39) averaged across three studies weighted by sample size.

In a study with a representative sample undergoing surgery, approximately 10% of the children fully met PTSD diagnosis, whereas 26.4% exhibited at least one symptom of each DSM-5 PTSD diagnostic criterion, and thus were designated partial PTSD (Ben-Ari et al., 2019c). In a more specific sample of children with congenital melanocytic nevi, the number of children with PTSD diagnosis was even higher (33.3%; Ben-Ari et al., 2019a).

Studies examining subthreshold PTSD found similar percentages of children exhibiting PTSS but not meeting diagnostic criteria of PTSD. For example, Connolly et al. (2004) found that 12% had symptoms that did not meet PTSD criteria. However, in a sample undergoing elective surgery, the total sample scored below the clinical cutoff of PTSD. PTSS of 92.3% of the children were in the “doubtful” intensity of the measure one month after surgery. Only 7.7% had mild PTSS one month postoperatively and 5.1% displayed mild PTSS 6 months later, demonstrating a notable decrease in symptoms (Ben-Amitay et al., 2006). A mild level of PTSD, was interpreted as not clinically significant, thus designated as subthreshold PTSD.

Two studies investigating the effects of intraoperative awareness among a small sample found that none of the children were in the clinically significant range of symptoms of posttraumatic stress (Lopez et al., 2008; Phelan et al., 2009).

#### Symptoms of Depression

Besides PTSD, only three studies explicitly examined depressive symptoms. Depressive symptoms prevalence rates ranged from 4% to 18.52%, with a weighted mean of 6.33% (*SD* = 5.1) weighted by sample size. Toren and Horesh (2007) reported 18.52% of children undergoing cardiac surgery displayed depressive symptoms. In contrast, in a study retrospectively examining behavioral functioning as measured by parental CBCL responses, no significant difference in affective problems on the DSM clinical scales between patients and controls emerged (Sarrechia et al., 2015). Similarly, another study found no significant difference between patient and control group in current and lifetime mood disorder diagnosis (DeMaso et al., 2014). In line with the reported studies above, Ben-Amitay et al. (2006) found depressive symptoms to be low (5% after one and 2.5% after six months) and marginally significantly decreasing at six months after surgery.

#### Symptoms of Anxiety

Another assessed outcome was symptoms of anxiety, which was examined by the same three studies, which also included depressive symptoms in their assessment. Anxiety symptoms prevalence rates ranged from 5% to 16.13%, with a weighted mean of 8.07% (*SD* = 4.43) weighted by sample size. Symptoms of anxiety were found to be rather low (12.5% after 1, and 10% after 6 months) and not significantly decreasing over time (Ben-Amitay et al., 2006). Similarly, current and lifetime anxiety disorder diagnosis did not significantly differ between the patients (5% and 12%, respectively) and the control group (7%, and 8%, respectively; DeMaso et al., 2014). This result was replicated in a study demonstrating no difference in anxiety problems assessed with the DSM clinical scales of the CBCL between patients and healthy controls (Sarrechia et al., 2015). In a study sample undergoing cardiac surgery, 16.13% endorsed sufficient symptoms to meet the criteria of a clinical anxiety disorder (Toren & Horesh, 2007).

#### Disruptive Behavior Disorder Symptoms

Symptoms of disruptive behavior disorder were reported in 16% of adolescents who have undergone arterial switch operation as infants (DeMaso et al., 2014). This made up the most common psychiatric diagnosis in their sample of adolescents.

#### Internalizing and Externalizing Symptoms

More broadly, five studies assessed behavioral symptoms with the CBCL, also phrased as a child’s overall distress level. The prevalence of children scoring in the clinical range reached from 8.1% to 45% with a weighted mean of 24.61% (*SD* = 8.28), whereas 3.8% to 26.7% of children were in the borderline range with a weighted mean of 8.31% (*SD* = 8.97). In general, internalizing symptoms were most frequently reported. A retrospective study reported in 45% of adolescents even several years after major neonatal surgery externalizing and internalizing symptoms (Kubota et al., 2011).

### Risk of Bias in Studies

The risk of bias scores for the studies included in this systematic review ranged from 4 to 7 out of 9 possible points (see Table S2 in the Supplementary Material). No study was considered to have low risk of bias. Four studies had 7 out of 9 points and were considered to have moderate risk of bias. Four studies had 6 out of 9 points, two studies had 5 out of 9 points, and one study had 4 out of 9 points. These seven studies were considered to have high risk of bias.

## Discussion

This is the first systematic review synthesizing the prevalence of psychological symptoms and disorders among children and adolescents after surgery. Eleven peer-reviewed heterogeneous studies were included to be as inclusive as possible in response to the understudied nature of this field. The majority of studies assessed PTSD symptoms and internalizing and externalizing symptoms as opposed to other psychological symptoms. Other studies in the injured or medically ill pediatric population have also commonly focused on PTSD (Rennick & Rashotte, 2009). Our findings indicate that there is an increased risk for PMTS for children and adolescents who undergo surgery as part of their medical conditions. A small but substantial part of children and adolescents after surgery endures psychological symptoms or disorders, such as posttraumatic stress, anxiety, depressive and behavioral symptoms. Prevalence rates suggest that most children and adolescents are resilient to the traumatizing effect of surgical procedures and adapt with some support which is concordant with previous findings (Le Brocque et al., 2020; Pinquart, 2020).

### Posttraumatic Stress Symptoms

Of the 11 included studies, eight studies reported on prevalence rates of PTSD symptoms after surgery. The averaged and weighted prevalence rate in the current review was approximately 13%, providing preliminary evidence that a small but substantial amount of children and adolescents endure posttraumatic stress after surgery. This aligns with meta-analytical findings reporting prevalence rates as high as 11.5% (Pinquart, 2020) and 12% (Kahana et al., 2006) after pediatric illness. Across other medical conditions, 10% to 20% develop PTSD (Forgey & Bursch, 2013).

The prevalence rates of PTSD within the included studies varied greatly from 0.0% to 33.3%. The great variation may derive from methodological factors (e.g., instruments for assessment of PTSD, cutoffs used), surgical factors (minor or elective surgery vs emergency surgery, length of hospitalization), the time elapsed after surgery, and cultural factors (e.g. country of study).

In addition, three studies reported on subthreshold PTSD symptoms (Ben-Amitay et al., 2006; Ben-Ari et al., 2019c; Connolly et al., 2004). Subthreshold PTSD rates ranged from 7 to 26% with an aggregated prevalence rate of 18.4%. Rates of subthreshold PTSD seem to be minimal higher compared with rates of PTSD diagnosis.

Three studies reporting low prevalence rates possibly account for an underestimation of the prevalence rate of PTSD. Firstly, Ben-Amitay et al. (2006) reported rather low rates of PTSS in children after elective surgery for which children might have been prepared. Secondly, after intraoperative awareness 0% of the children developed PTSD (Lopez et al., 2008; Phelan et al., 2009) while psychological sequelae were observed in adults (Vulser et al., 2015). Psychological symptoms in children who could not be followed up (attrition rate of 36.36% and 42.86%) could not be excluded since parents reported family issues when called.

### Symptoms of Depression and Anxiety

The averaged and weighted prevalence rate each across three studies was 6.33% for postoperative depressive symptoms and 8.07% for anxiety symptoms. This mirrors pediatric studies reporting 7% to 16% anxiety symptoms after burn-injury and meningococcal disease (De Young et al., 2012; Shears et al., 2007) and 7% to 17% depressive symptoms after critical illness or injury (Davydow et al., 2010; Kassam‐Adams et al., 2015b; Zatzick et al., 2008). Rates for depressive and anxiety symptoms seem to be a little smaller compared with PTSS.

It is important to point out that depression, anxiety, opposing behavior, and posttraumatic stress symptoms may occur comorbidly (De Young et al., 2012; Kassam‐Adams et al., 2015b). In the included study by Toren and Horesh (2007), 33% of the PTSS subgroup scored above the clinical cutoff for anxiety symptoms. In contrast, in the non-PTSS subgroup, only 9.1% scored above the cutoff for anxiety symptoms. Similarly, 29% and 15% of adolescents in the PTSS and non-PTSS subgroups, respectively, were at risk for depressive disorder. However, comorbidity was not analyzed explicitly in any of the included studies.

### Internalizing and Externalizing Symptoms

Internalizing symptoms and externalizing symptoms were measured by means of the Child Behavior Checklist (CBCL) in five studies. Almost one fourth (24.61%) of parents reported internalizing and externalizing symptoms in their children and adolescents after surgery. This finding aligns with a study that demonstrated that 25.9% of critically ill children reported internalizing and externalizing symptoms as response to hospitalization (Melnyk et al., 2004). Of the included studies, Kubota et al. (2011) found that almost one half of children and adolescents who had undergone major neonatal surgery display behavior symptoms even several years after the event, suggesting long-term impairment of neonatal surgery. Moreover, aggregated 8.31% were in the borderline range of the CBCL questionnaire. Taken together, approximately 35% display behavior symptoms that may disrupt functioning and development.

### Disruptive Behavior Disorder Symptoms

Only one study assessed more broadly psychiatric diagnoses, including mood, anxiety, and disruptive behavior disorders in a specific population of adolescents with d-transposition of the great arteries who had arterial switch operation as infants (DeMaso et al., 2014). The most common psychiatric diagnosis was ADHD. It was present in 16% of adolescents in the patient group even up to 16 years after surgery for congenital heart disease. In contrast, only 3% of the control group was diagnosed with ADHD.

### Methodological Quality of the Included Studies and Implications for Research

The majority of studies were considered to be at high risk of bias. Notably, most studies were prospective, which can be less prone to bias compared to retrospective studies. Heterogeneity between studies derived from the age range included, variation of sample size from 4 to 139, and variation in time of assessment of psychological outcomes. All studies used valid and reliable instrument. However, different instruments restrict homogeneity of the included studies and the focus on posttraumatic stress may account for an underestimation of PMTS. Thus, a standardized instrument assessing the broad psychological sequelae of medical trauma might be of interest to researchers and clinicians as well.

Two studies included a healthy control group (DeMaso et al., 2014; Sarrechia et al., 2015). Thus, differentiating whether the medical condition that required surgery or the surgery itself elicited psychological consequences is difficult. Future studies could compare PMTS between surgical and nonsurgical groups which would allow to conclude on the effect of surgical factors. In the surgery group the medical condition necessitates surgery whereas the nonsurgical control group does not undergo surgery for the same underlying medical condition. Admittedly, duration of hospitalization, severity of illness and number of procedures were found to associate with PTSS (Connolly et al., 2004; Rennick et al., 2002). However, meta-analyses indicate a low predictive value of objective characteristics whereas subjective experience and perceived life-threat are among the strongest predictors of PTSS (Cox et al., 2008; Trickey et al., 2012). From a psychological perspective, pediatric otorhinolaryngology and extremity fracture which are often treated surgically as well as conservatively might be suitable. In cooperation with medical experts, an illness or injury appropriate for that investigation needs to be confirmed.

Further, only two included studies examined psychological symptoms after surgery in children younger than 6 years of age (Ben-Ari et al., 2018, 2019a) resulting in an underestimation. Despite difficulties to assess psychological consequences (Davydow et al., 2010), widespread adverse psychological consequences after invasive medical procedures were reported for the youngest (De Young et al., 2012; Graf et al., 2011). More research efforts in projects like the “the caring intensively study" (Rennick et al., 2014) or a recent qualitative analysis (Lopez et al., 2019) need to be devoted to this age range.

Results should be replicated in greater representative samples of general surgery wards a few months after the event. Different psychological symptoms after different kind of surgeries should be assessed to counteract the underestimation of PMTS after surgery.

### Strengths and Limitations of the Current Review

The findings should be interpreted within the context of several limitations deriving mostly from study eligibility criteria and the included studies. Even though we are confident that our protocol-driven and complementary search approach yielded most of the published studies to date, the review lacks a systematic search for unpublished studies. On the one hand, the review is prone to "publication bias" (Rosenthal, 1995) since non-significant results could be disregarded. On the other hand, peer-reviewed studies ensure a certain level of quality.

Heterogeneity *within* studies concerning the type of surgical procedure and the percentage of children not undergoing surgery limits results. No restriction was placed on the kind of surgery leading to the inclusion of studies on elective and emergency surgery as well as on intraoperative awareness. The heterogeneity *between* studies assures high generalizability across pediatric surgical intervention. When calculating prevalence rates, weighting did not consider methodological heterogeneity and heterogeneous surgical events but was based on the sample size contributing to the validity of the review and being a strength. Nonetheless, methodological heterogeneity and heterogeneous surgical events were not considered, thus limiting the validity of the reported prevalence rates. However, data of the included studies did not allow for examination of the size of between-group differences by study characteristics, such as type of illness, or duration of hospitalization. Future research might want to answer questions regarding correlates of PTSS and psychological symptoms in children and adolescents after surgery with meta-analytic strategies.

### Clinical Implications

The implications of this systematic review are manifold. Training for physicians, pediatricians, and hospital staff should be improved regarding detection and diagnostics of psychological symptoms and disorders despite individual and developmental differences in symptom expression of PTSD. Moreover, the psychosocial competence of hospital staff is fundamental for operative support. Medical or so-called ward psychologists should work together with trained hospital staff. In Germany psychologists assess psychological symptoms in intensive care patients and provide psychological support for patients and relatives. However, only a few hospitals can implement professional psychological care (Deffner et al., 2021). According to the guidelines by the Association of the Scientific Medical Societies in Germany targeting pediatric oncology, intrusive surgical procedures call for psychosocial treatment (Arbeitsgemeinschaft der Wissenschaftlichen Medizinischen Fachgesellschaften [AWMF], 2019). However, recommendations by professional associations concerning pediatric surgical procedures in general do not exist.

Aside from improved training, consequent early screening and prevention and psychoeducation should be applied routinely. Sharing information about the medical condition with children (Ben-Ari et al., 2019b), disclosure about expected pain, and enabling the child to decide and to choose (Ahrens-Eipper & Nelius, 2017) show ways to reduce posttraumatic distress. Coping strategies such as distraction and relaxation should be supported (Forgey & Bursch, 2013). If possible, the duration of invasive treatment and PICU stay should be reduced (Rady et al., 2020). Surgeons could engage in working relationships with parents (DeMaso & Snell, 2013). During preparatory surgical consultations parents should be informed about the risk, the different expressions of psychological symptoms and resources.

## Conclusion

The present study aimed to systematically review studies on pediatric medical traumatic stress (PMTS) following surgical procedures in children and adolescents. There is a large body of research on pediatric illness and injury while studies on PMTS after surgery remain scarce. This is the first systematic review on the prevalence of psychological consequences among children and adolescents after surgery, including posttraumatic symptoms, anxiety and depressive symptoms, and internalizing and externalizing symptoms. Due to the potentially traumatizing situations faced by children who undergo surgery, it is important to provide an overview of existing research in this understudied area.

Our findings show that a small but substantial number of children exhibit posttraumatic stress symptoms, anxiety, depressive, internalizing and externalizing symptoms after surgery. Respective prevalence rates are concordant with rates from other pediatric populations. However, prevalence rates may underestimate the actual number due to the lack of studies focusing on heterogeneous psychological consequences and a limited age range.

In conclusion, medical trauma resulting from pediatric surgery should be recognized by clinicians providing sophisticated care for children. Apart from better training for clinicians, incorporation of prevention, early psychological screening and referral to psychosocial care are recommended. Future research should improve methodological limitations (e.g., control group, age range), replicate existing findings and provide a broader base of evidence.

## Supplementary Information

Below is the link to the electronic supplementary material.

Supplementary file1 (DOCX 21 KB)

## Footnotes

1 the terms surgery, surgical procedure, and operation, are used interchangeably from a medical perspective.

## Declarations

All data files, associated codebooks, and analysis scripts are publicly available on the study’s page on the Open Science Framework: https://osf.io/vp9d8/.

Idea/ concept for study design: Susan Sierau, Background/rationale/significance: Susan Sierau and Anna Stanzel, Literature review/search: Anna Stanzel, Design: Susan Sierau and Anna Stanzel, Statistical analysis: Anna Stanzel, Writing part or all of the content: Anna Stanzel, Critical revision of manuscript: Susan Sierau and Anna Stanzel, Editing of manuscript: Susan Sierau and Anna Stanzel, Preparation of tables/figures: Anna Stanzel
