# Supplementary material for: Pediatric Medical Traumatic Stress (PMTS) following Surgery in Childhood and Adolescence: a Systematic Review
Source: J Child Adolesc Trauma. 2021 Aug 21;15(3):795–809. doi: 10.1007/s40653-021-00391-9 (PMC9360277; doi:10.1007/s40653-021-00391-9)
Supplement: Supplementary file 1 — Supplementary file1 (DOCX 21 KB) [file 40653_2021_391_MOESM1_ESM.docx]

**Supplementary Material**

Table S1
*Database Search Limiters*

| Category | PsycInfo | PubMed |
| --- | --- | --- |
| Type of Studies | Peer-Reviewed | Option not available |
| Age Groups | Childhood: birth-12 years  Neonatal: birth-1 months  Infancy: 2-23 months  Preschool Age: 2-5 years  School Age: 6-12 years  Adolescence: 13-17 years  Young Adulthood: 18-29 years | Child: birth-18 years |
| Language | English, German | English, German |
| Expanders | Apply equivalent subjects | NA |
| Search modes | Boolean/Phrase | Boolean/Phrase |

Table S2
*Risk of Bias Tool*

| **Study ID** | **Item 2** | **Item 3** | **Item 4** | **Item 5** | **Item 6** | **Item 7** | **Item 8** | **Item 9** | **Item 10** | **Summary item on the overall risk of study bias ^a^** |
| --- | --- | --- | --- | --- | --- | --- | --- | --- | --- | --- |
| ben-amitay2006 | 0 | 0 | 1 | 1 | 1 | 1 | 1 | 1 | 1 | 7 |
| ben-ari2018 | 1 | 0 | 1 | 0 | 1 | 1 | 1 | 1 | 1 | 7 |
| ben-ari2019a | 0 | 0 | 0 | 1 | 1 | 1 | 1 | 1 | 1 | 6 |
| ben-ari2019b | 1 | 0 | 1 | 0 | 1 | 1 | 1 | 1 | 1 | 7 |
| connolly2004 | 0 | 0 | 0 | 1 | 1 | 1 | 1 | 1 | 1 | 6 |
| demaso2014 | 0 | 0 | 1 | 1 | 1 | 1 | 1 | 1 | 1 | 7 |
| kubota2011 | 0 | 0 | 0 | 0 | 1 | 1 | 1 | 1 | 1 | 5 |
| lopez2008 | 0 | 0 | 0 | 0 | 1 | 0 | 1 | 1 | 1 | 4 |
| phelan2009 | 0 | 0 | 0 | 1 | 1 | 0 | 1 | 1 | 1 | 5 |
| sarrechia2015 | 0 | 0 | 1 | 0 | 1 | 1 | 1 | 1 | 1 | 6 |
| toren2007 | 0 | 0 | 0 | 1 | 1 | 1 | 1 | 1 | 1 | 6 |

*Note*. Review author’s judgment about each risk of bias item for each included study

^a^ indicates low risk of bias: 8 or 9; moderate risk of bias: 7; high risk of bias: 6 or less
